# Proteoglycans in the Pathogenesis of Hormone-Dependent Cancers: Mediators and Effectors

**DOI:** 10.3390/cancers12092401

**Published:** 2020-08-24

**Authors:** George Tzanakakis, Eirini-Maria Giatagana, Andrey Kuskov, Aikaterini Berdiaki, Aristidis Tsatsakis, Monica Neagu, Dragana Nikitovic

**Affiliations:** 1 Laboratory of Histology-Embryology, School of Medicine, University of Crete, 71003 Heraklion, Greece; tzanakak@uoc.gr (G.T.); Eirini_gt@hotmail.com (E.-M.G.); berdiaki@uoc.gr (A.B.); 2 Laboratory of Anatomy, School of Medicine, University of Crete, 71003 Heraklion, Greece; 3 Department of Biomaterials, D. Mendeleev University of Chemical Technology of Russia, Miusskaya sqr. 9, 125047 Moscow, Russia; a_n_kuskov@mail.ru; 4 Laboratory of Toxicology, School of Medicine, University of Crete, 71003 Heraklion, Greece; tsatsaka@uoc.gr; 5 Department of Immunology, Victor Babes National Institute of Pathology, 050096 Bucharest, Romania; neagu.monica@gmail.com

**Keywords:** proteoglycans, hormone-dependent tumors, breast cancer, prostate cancer, tumor microenvironment, tumor biology, immunosurveillance

## Abstract

Hormone-dependent cancers exhibit high morbidity and mortality. In spite of advances in therapy, the treatment of hormone-dependent cancers remains an unmet health need. The tumor microenvironment (TME) exhibits unique characteristics that differ among various tumor types. It is composed of cancerous, non-cancerous, stromal, and immune cells that are surrounded and supported by components of the extracellular matrix (ECM). Therefore, the interactions among cancer cells, stromal cells, and components of the ECM determine cancer progression and response to therapy. Proteoglycans (PGs), hybrid molecules consisting of a protein core to which sulfated glycosaminoglycan chains are bound, are significant components of the ECM that are implicated in all phases of tumorigenesis. These molecules, secreted by both the stroma and cancer cells, are crucial signaling mediators that modulate the vital cellular pathways implicated in gene expression, phenotypic versatility, and response to therapy in specific tumor types. A plethora of deregulated signaling pathways contributes to the growth, dissemination, and angiogenesis of hormone-dependent cancers. Specific inputs from the endocrine and immune systems are some of the characteristics of hormone-dependent cancer pathogenesis. Importantly, the mechanisms involved in various aspects of cancer progression are executed in the ECM niche of the TME, and the PG components crucially mediate these processes. Here, we comprehensively discuss the mechanisms through which PGs affect the multifaceted aspects of hormone-dependent cancer development and progression, including cancer metastasis, angiogenesis, immunobiology, autophagy, and response to therapy.

## 1. Introduction

All tumor types develop a unique tumor microenvironment (TME) that boasts different compositions of cancerous, non-cancerous, stromal, and immune cells in each phase of cancer progression. The different cell subtypes of TME interact with each other but also with components of the extracellular matrix (ECM) surrounding the cells [1]. The ECM is a crucial regulator of all cellular functions and a significant component of the TME. Importantly, ECM cues coordinate the different effectors of the TME and modulate the plethora of signaling pathways involved in the pathogenesis of cancer [2,3]. Even early reports showed that desmoplasia, or an accumulation of the ECM, is a characteristic property of tumors, and increased ECM contents are frequently associated with dismal prognosis in various tumor types [4]. Proteoglycans (PGs) are significant components of the ECM implicated in all phases of tumorigenesis. Their “hybrid” composition, consisting of a protein core and glycosaminoglycan (GAG) chains, bestows these molecules with high versatility and ability to interact with many cellular effectors [5]. Modifications in PG content and structure are correlated with disease progression in various cancer types. Importantly, PGs, like other components of the ECM, are secreted by both stroma (e.g., cancer-associated fibroblasts) and cancer cells [6]. Of note, PGs are crucial regulators of the bioavailability of growth factors, hormones, and cytokines as well as the resulting activation of their respective receptors that modify gene expression, phenotypic versatility, and response to therapy in specific tumor types [7]. Recent advances in omics technologies have shown that PGs are among the molecules whose gene signature is predictive of cancer development and prognosis [8].

Hormone-dependent cancers exhibit high morbidity and mortality. In spite of advances in therapy, the treatment of hormone-dependent cancers remains an unmet health need. Hormones are vital signaling molecules that are produced by glands and play a crucial role in regulating body physiology and pathophysiology [9]. These active mediators, such as androgens and estrogens, can control cell behavior by binding to specific receptor proteins in the target cell [10]. Their critical role in cell signaling gives hormones the ability to deregulate the functions of target cells under certain conditions and, thus, to promote a cancerous phenotype.

Two of the most common solid malignancies that are sex- and hormone-dependent are breast cancer (BC) and prostate cancer (PC). A plethora of deregulated signaling pathways contributes to the growth, dissemination, and angiogenesis of these tumors [11,12].

Various mechanisms have been found to be correlated to resistance in hormone-dependent cancers, with specific differences exhibited between BC and PC. There is evidence that immunological responses to foreign and self-antigens are sex-dependent, and there are differences in innate and adaptive immune responses. These sex hormone-related changes to immunity may be associated with different immunoediting in hormone-dependent cancer and explain the differential susceptibility of males and females to malignancies [13,14]. Autophagy and apoptosis have been correlated to chemoresistance and cancer stem cell (CSC) properties [15,16]. Importantly, the mechanisms involved in various aspects of cancer progression are executed in the ECM niche of the TME, and the ECM components crucially mediate these processes. Here, we comprehensively discuss the mechanisms through which PGs affect the multifaceted aspects of hormone-dependent cancer development and progression. Determining the individualized role of PGs in cancer patients can lead to new therapeutical strategies in the form of adjuvants or treatments to replace the standard therapy protocols.

## 2. Proteoglycans

PGs are composed of a protein core into which one or more GAG chains are covalently bound [17]. The GAG chains consist of repeated disaccharide units and are negatively charged [18]. The disaccharide building blocks consist of an amino sugar (glucosamine that is N-acetylated or N-sulfated or N-acetylgalactosamine) and uronic acid (glucuronic acid or iduronic acid) or galactose [5]. There are four different types of GAG chains, usually glycosylate PGs, e.g., heparan sulfate (HS), chondroitin sulfate/dermatan sulfate (CS/DS), or keratan sulfate (KS) [17]. Serglycin is the only known PG which can be decorated with the GAG heparin [19]. CS/DS, HS, and heparin bind to the protein core through a specific trisaccharide linker composed of two galactose (Gal) and one xylose (Xyl) residue. The linker explicitly binds to a serine residue of the protein core via an O-glycosidic bond, creating the final structure GAG-GalGalXyl-O-CH2-protein [20].KS is a sulfated poly-N-acetylgalactosamine chain substituted on a limited number of PGs as an N-linked or O-linked chain [5].The type of GAG substitution classifies the 45 PG members as chondroitin sulfate PGs (CSPGs), heparan sulfate PGs (HSPGs), or keratan sulfate PGs (KSPGs). The properties of PGs are dependent on both the protein core and the GAG substitution [5,17,21].

Moreover, PGs can be glycosylated by more than one kind of GAG [22,23]. GAG chains can interact with a wide variety of signaling macromolecules to regulate fundamental biological processes [20] and, thus, bestow PGs with a plethora of structural and functional variations [18]. The glycosylation of PGs is initiated in the endoplasmic reticulum, further orchestrated in the Golgi apparatus, and is mostly mediated by glycosyltransferases that reside in this complex organelle [24].

The PGs can be classified based on their location as intracellular (e.g., serglycin), at the cell membrane (e.g., betaglycan), pericellular (e.g., perlecan), and away from the cell or “extracellular” (e.g., biglycan). The small leucine-rich proteoglycans (SLRPs) are a prominent component of extracellular PGs, comprising the largest class of PGs [22]. Serglycin is the only intracellular PG, initially identified in granules of mast cells, where it stabilizes proteases that are released upon inflammation [25]. However, in continuation, it was shown that the majority of immune cells express serglycin and store it within intracytoplasmic granules to control the bioactivity of various inflammatory mediators like chemokines or cytokines [26].

Hyalectans are another family of extracellular PGs. This family compromises four PGs, namely aggrecan, versican, neurocan, and brevican, which are endowed with distinct similarities at both the genomic and protein levels [27]. All four hyalectan members share a tri-domain structure consisting of an N-terminal domain with affinity towards hyaluronan, a central domain decorated with CS chains, and a C-terminal region with an ability to bind lectins [27]. Due to alternative splicing and variability in the level of glycosylation, these PGs exert different operating properties with the ability to function as molecular bridges between cell surfaces and extracellular molecular networks [17]. Aggrecan, in common with other hyalectan members, has the ability to aggregate into large supramolecular complexes and is the principal load-bearing PG component of cartilage [28]. Versican is the largest hyalectan member when expressed as the full-length isoform V0 [27]. Versican isoforms, the result of discrete splicing, exhibit high variability in tissue expression correlated to complex roles in vital cellular functions, including adhesion, migration, and inflammation [29]. Neurocan and brevican are important hyalectans of the brain involved in the regulation of neural axon outgrowth and the biology of neural stem cells, among other processes [30,31,32,33].

As regarding cell surface PGs, thirteen genes encode these proteins. Of these, seven are transmembrane PGs, e.g., syndecans, while the remaining six are glypicans, glycosyl phosphoinositol (GPI)-anchored PGs [17]. These PGs mainly contribute to cell migration and adhesion processes as their intracellular domain connects the cytoskeleton and kinases with the extracellular environment [34]. Moreover, transmembrane PGs interact with or serve as coreceptors for many signaling molecules, such as growth factors or Wnt proteins. In this manner, they have the ability to enhance or inhibit signaling pathways, affecting their respective cell functions [35,36]. Pericellular PGs are also found anchored on the cell surface of different cell types via integrins or cell surface receptors. These PGs are mostly HSPGs, and their HS chains can be cleaved by various enzymes and act as pro-angiogenic factors [37]. The largest PG category is the extracellular PGs, which are structural components of the ECM [17] that have a supportive role for the cells in tissues, but also act as signaling mediators.

SLRPs are defined as PGs with a relatively small protein core (36–42 kDa) harboring multiple leucine-rich repeats (LRRs) and substituted with GAG chains [38]. Initially, the SLRPs were classified into three distinct classes based on the conservation of the amino acid residues of their protein cores, the organization of disulfide bonds at the N- and C-terminal regions of the molecule, and their genomic organization/gene homology [39,40,41]. In continuation, the SLRPs gene family has been expanded to include 18 genes classified into five separate subfamilies [42]. Many studies have shown that these PGs bind with biologic mediators, including growth factors, to participate in cells’ interactions with their microenvironment [43,44]. Indeed, the SLRPs act as signaling molecules that participate in the regulation of basal cellular functions, like proliferation, migration, and differentiation. The SLRPs can interact with tyrosine kinase receptors, and this is one of their primary mechanisms of action through which they regulate downstream intracellular signaling and, ultimately, cellular behavior [45,46,47,48].

## 3. Proteoglycan Expression in Hormone-Dependent Cancer

It is well established that PGs take part in signaling pathways that control cellular functions. PGs’ expression and glycosylation profiles differ between normal and malignant tissues, a fact that affects cell behavior and differentiation status.

The majority of BC cells express estrogen receptor-α (ER-α), a diagnostic biomarker for this malignancy, while the role of the second ER isoform, ERβ, expressed by the mammary gland, is yet to be defined in BC. Estrogens regulate the functions of the female reproductive system by binding to these specific receptors [49]. ER-α is also expressed by cancerous prostate cells, while, respectively to estrogens, androgens are the male sex hormones that bind to androgen receptors (AR) and are expressed during all stages of prostate carcinogenesis and related to hereditary PC [50,51].

PGs show discrete expression patterns between normal and tumor tissues. Thus, Suhovskih et al. showed that the expression profiles of PGs glypican-1, perlecan, syndecan-1, aggrecan, versican, NG2, brevican, decorin, and lumican differ between healthy prostate tissue and prostate tumors [52]. Indeed, it was demonstrated that versican, decorin, and biglycan were prevalent PGs in normal prostate tissue stroma, whereas syndecan-1 and glypican-1 were localized to the epithelial component. In prostate tumor tissues, a marked attenuation of total decorin and lumican expression was evident, whereas syndecan-1 and glypican-1 expression was increased in tumor stroma and attenuated in tumor epithelial cells [52]. Indeed, the downregulation of syndecan-1 expression in PC was associated with the aggressive behavior of these cells [53] and clinical tumor progression [54]. Syndecan-2 was found to be expressed by PC cells and not expressed by normal prostate epithelial cells [55]. Syndecan-1 and -2 expression by PC cells was proposed as a useful prognostic marker for patients who presented with clinically localized cancer. In combination with prostate-specific antigen, evaluation of the expression of these PGs resulted in improved assessment of the risk of recurrence [56].

On the other hand, the expression of lumican in the stroma circumjacent to primary prostate tumors was found to attenuate the progression of this malignancy [57]. Recently, it was shown that the SLRP fibromodulin has discrete expression among PC and benign tissues [58]. PC tissues express this PG, as do PC cell lines with, interestingly, the highest expression determined in the androgen-sensitive human metastatic prostate adenocarcinoma LNCaP clone [59]. Moreover, the glypican-1 expression in urine cell sediments was proposed as a possible positive marker for PC [60]. The low expression of another glypican member, glypican-5, has, on the other hand, been correlated to PC progression [61].

It is worth noting that early studies have shown that androgens affect the expression of PGs in the prostate gland [62], and that the effects differed between wild type and castrated rats [63]. These authors suggested that the effects of androgens on prostatic growth are partly dependent on the interactions between the parenchyma and stroma facilitated by PGs [63]. Moreover, the GAG component differs among benign and malignant prostate tissues, and GAGs, along with other glycan types, have been suggested as biomarkers for PC [64]. Edwards, in 2012, discussed that in PC, decorin, which also interacts with TGF-b [65,66], and betaglycan inhibit tumor growth and metastasis, while versican and perlecan respectively promote cell motility and invasion, and tumor cell growth and angiogenesis.

Indeed, it is suggested that PGs are a significant venue of communication between normal prostate epithelial cells and fibroblasts, and changes in PG expression by cancer cells contribute to the deregulation of cell–cell contact growth inhibition [67].

PGs have likewise been indicated as significant mediators of BC tumorigenesis [68]. Mammographic density mirrors alterations in the composition of breast tissue as distinguished on a mammogram and is immediately correlated to the dominance of epithelial and stromal components [69]. Histologically, the stroma is the region exhibiting the highest mammographic density [70]. Importantly, mammographic density is immediately associated with the protein, including PG content, of the breast tissue [71]. PGs are discretely expressed in normal breast tissue depending on hormone status, whereas there are significant alterations of PGs in BC tissues. One example is the pericellular PG perlecan, whose expression is significantly attenuated in BC compared with in healthy tissue [72]. These changes in mammographic density, dependent on PGs, are attributed to their ability to retain water [73].

HSPGs participate in the development of the mammary gland, during which substantial changes in mammographic density are noted [74]. Indeed, mammographic density is an essential independent BC risk factor and is correlated to other various BC risk factors. Therefore, Hanna and Diorio suggest that it is significant to examine the correlation of the expression of individual proteins with changes in mammographic density and evaluate their correlation with BC risk [69]. The expression pattern of syndecan-1 is altered during BC tumorigenesis [75]. It is expressed in the stroma of the BC and by the epithelium of normal breast tissue [76]. Indeed, it is suggested that the redistribution of syndecan-1 leads to significantly increased mammographic density, and its total expression is higher in the dense as compared to non-dense breast tissue of postmenopausal women [77]. Moreover, the expression of syndecan-1 was found to be lower in normal breast tissue during luteal as compared to the follicular phase of the menstrual cycle [78]. These data demonstrate that the expression of syndecan-1 is directly dependent on estrogen levels, which increase during the follicular phase of the menstrual cycle and are correlated to the transient changes in the density of breast tissue stroma [78].

Syndecan-2 was found to be expressed by BC cell lines [79] and to facilitate the ability of BC cells to invade [80]. The expression of syndecan-4 seems to attenuate the aggressive behavior of BC cells as it neutralizes the pro-invasive properties of syndecan-2 [81].

The expression of glypicans is also modified in BC tissues. Indeed, the expression of glypican-1 is found to be significantly upregulated in BC and was determined to affect the action of heparin-binding growth factors [82]. By contrast, glypican-3 is downregulated [83] and attenuates the metastasis of BC cells in a syngeneic BC model [84].

The expression of the class I SLRPs biglycan and decorin as well as of enzymes implicated in PGs synthesis was found to be modified in BC. Indeed, the unique identified pattern of their expression was determined to have clinical relevance [85]. An early report, however, suggested that the expression of biglycan is low in both normal and BC tissues [86]. Interestingly, the expression of biglycan was shown to be enhanced in precancerous lesions [87].

Lumican, an SLRP also implicated in tumorigenesis [7], is the main SLRP expressed by normal and cancerous breast tissue [88]. The effects of lumican on BC pathogenesis are controversial. Early reports indicate that lumican is mostly expressed by BC stroma cells and was positively correlated with higher tumor grade, lower expression of ERs, as well as to the younger age of patients [89]. Furthermore, an increased expression of lumican and decorin correlated to enhanced mammographic density was shown in benign and precancerous breast lesions [90]. Specific lumican polymorphisms were associated with BC risk. Thus, LUM rs2268578 was correlated with ER-positive BC in the Mayo Clinic study, whereas a modest association was detected in the Studies of Epidemiology and Risk Factors in Cancer Heredity SEARCH sample [91].

**Figure 1:**
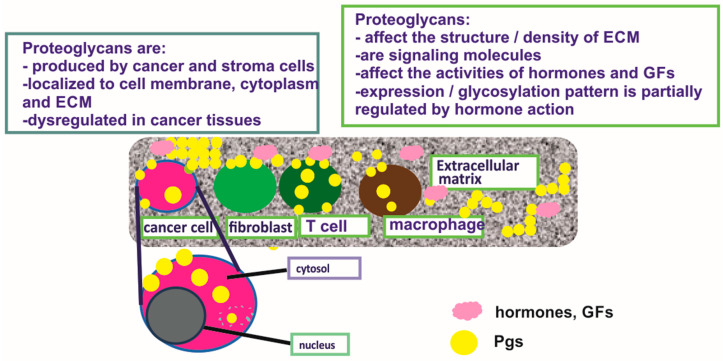
The expression and critical roles of proteoglycans (PGs) in hormone-dependent cancer. PGs are expressed at the cell membrane and cytosol of cancer and stroma cells and secreted to the extracellular matrix (ECM). They contribute to the structure of cancer tissues, e.g., the regulation of tissue density and affect the activities of growth factors and hormones.

BC tissues express the large CS-containing PGs, hyalectans such as versican [92]. Indeed, all known isoforms of versican are overexpressed in the malignant lesions, both at the protein and mRNA levels [93]. These authors had also detected a novel alternatively spliced versican isoform (V4) whose expression was enhanced in human BC [93]. A separate study showed high versican expression in the perilesional stroma of a specific subclass of ductal in situ carcinomas, and that this expression pattern was correlated to the high grade (G3) category of the tumor [94]. Canavese et al., however, did not detect changes in the expression of versican in lobular in situ carcinoma as compared to normal breast tissue [94]. Furthermore, it was shown that versican levels were increased in cancer tissues exhibiting malignant-appearing microcalcifications as compared to normal breast tissues. On the other hand, the hyalectan, aggrecan transcription levels were not altered in BC tissues [95]. The expression and critical roles of PGs in hormone-dependent cancer are concisely depicted in Figure 1.

## 4. Role of PGs in Hormone-Dependent Cancer Growth, Metastasis, and Angiogenesis

### 4.1. Effects of PGs on Cancer Cell Growth and Epithelial-to-Mesenchymal Transition (EMT)

It is well established that cancer cells acquire abnormal behavior during the multistep development of human tumors. Cancer cells present uncontrolled proliferation as compared to the regulated growth of cells in normal tissues, and they activate invasion and metastasis mechanisms, as many signaling pathways are suppressed or overactivated. Furthermore, cancer cells create a tumor microenvironment which contributes to the acquisition of these features. Several PGs and GAGs are misexpressed in cancers, acting as prognostic markers and contributing to tumor progression via the modulation of various “Hallmarks of Cancer” [96]. Cancer stem cells (CSCs; or tumor-initiating cells) are characterized by self-renewal, unlimited proliferation, and active DNA repair mechanisms, which lead to apoptosis resistance and de-differentiation mechanisms. Due to these properties, CSCs display resistance to therapies or they evade the immune system, resulting in tumor progression [97]. Many studies show that PGs, through their GAG components, regulate CSC phenotypes. HS chains that are bound onto the cell surface and ECM PGs can regulate the biological functions of BC cells due to their specific binding affinities towards a plethora of matrix molecules, including growth factors and molecules that mediate inflammation [98]. Other studies indicate that HSPG expression levels and their substitution with GAG chains play an essential role in the maintenance of self-renewal of pluripotent cells and are crucial for determining a specific cell fate [99,100,101]. Estrogens can affect the expression of ECM components and, thus, modulate the progression of uterine leiomyoma [102]. Furthermore, hormonal therapy resistance is closely related to the cancer stem cell-like properties of luminal breast cancer [103].

The process of epithelial-to-mesenchymal transition (EMT) modifies epithelial polarity and prevents cell–cell adhesion. Tumor cells acquire an SC-like phenotype, enhancing their migratory and invasive properties. As cells undergo EMT states, they acquire different functional characteristics, such as increased proliferation, propagation, invasion, and metastasis [104], which can be regulated by PGs.

PGs participate in BC cell EMT transformation, as demonstrated by a study utilizing BC cell lines of different phenotypes [105]. MCF-7 cells, which exhibit epithelial characteristics, and MDA-MB-231, a more aggressive BC cancer cell line with mesenchymal characteristics, were used to study the role of syndecan-1. This PG regulated stemness-associated pathways through an axis comprised of IL-6, the IL-6 receptor sIL-6R, and the chemokine CCL20 [105]. Furthermore, syndecan-1 facilitated the activation of STAT-3 and NF-kB transcription factors by upregulating the expression of the coreceptor for Wnt signaling, LRP-6. These pathways were shown to enhance the formation of tumor spheres by MCF-7 BC cells grown in suspension culture [105]. Downregulation of this PG expression resulted in the reduction of stemness-related phenotypic characteristics of MDA-MB-231 cells, indicating that syndecan-1 enhances EMT in breast cancer [105].

On the other hand, serglycin regulates the secretion of IL-8 in MCF-7 cells independently of their ERα status, and promotes their proliferation, migration, and invasion by triggering downstream IL-8/CXCR2 signaling cascades. This autocrine activation of the IL-8/CXCR2 signaling axis causes increased expression of mesenchymal markers vimentin and fibronectin, and the EMT-related transcription factor Snail2, which resulted in enhanced EMT of MCF-7 BC cell line [106].

The HSPGs syndecan-1 and -2 are discretely expressed among benign and of different Gleason score malignant prostate tissue samples. The alterations in the expression patterns of these PGs, in parallel with E-cadherin and β-catenin, suggests that they may participate in the EMT and the progression of PC [107]. Another HSPG, glypican 5, negatively regulates PC proliferation and invasion. It also inhibits EMT and Wnt signaling pathway activation, which are controlled by the SP-1 transcription factor [108].

### 4.2. SLRPs—Unique Regulators of Cancer Cell Functions

Numerous studies show the significant role of SLRPs in controlling cell signaling that is associated with tumorigenesis [7,43]. Lumican is the major SLRP of normal breast tissue and is variably expressed in benign and malignant breast lesions. This SLRP effectively regulates ER expression in BC cells, affecting their behavior and attenuating specific cell functions, like proliferation, migration, and invasion [109]. Besides, it was recently shown that lumican enhances the expression of α2 and β1 integrin subunits in aggressive ERβ-positive MDA-MB-231 and transfected ERβ-suppressed shERβMDA-MB-231 cells in comparison to ERα-positive MCF-7/c cells of low invasive capability. Moreover, lumican exerted discrete effects on α1, α2, α3, αVβ3, and αVβ5 expression among these cell lines, suggesting an essential role for ERβ in affecting the expression levels of adhesion molecules. These changes increased the ability of the highly metastatic MDA-MB-231 cells to attach to collagen. Furthermore, the migration of cells treated with lumican was downregulated due to attenuation of downstream signaling pathways, including FAK (focal adhesion kinase), ERK1/2 MAPK 42/44, and Akt [110]. Lumican inhibits expression of cortactin and MMP-14 that are responsible for the formation and functions of invadopodia, which facilitate the invasion and metastasis of mesenchymal cells. This inhibition transforms BC cells of the mesenchymal phenotype into epithelial-like cells with a lower metastatic capacity [110]. Indeed, Karamanou et al. suggest that the antitumor effects of lumican can partly be attributed to the above signaling mechanisms [110].

The role of biglycan in tumorigenesis is controversial, with most reports attributing oncogenic properties to this PG. The upregulation of biglycan has been reported in many malignancies, such as ovarian cancer [111], PC [112], and osteosarcoma [47]. On the other hand, biglycan expression is low in BC [86]. The overexpression of this SLRP is associated with aggressive growth and metastasis of ovarian cancer [111] and osteosarcoma [47] as well as with the poor prognosis of PC patients [112]. These effects are mediated through different signaling pathways. Thus, biglycan regulates the activation of FAK, which modifies cell adhesion and motility by transmitting ECM signals via cell membrane integrins to the cytoplasm [113]. FAK activation leads to the phosphorylation of Tyr residues, after which FAK binds Src and is further phosphorylated, promoting a MAPK-associated angiogenic switch during tumor progression [114]. Furthermore, this signaling promotes cancer cell proliferation and migration [114]. It is suggested that biglycan-dependent FAK signaling activation due to biglycan overexpression leads to increased matrix stiffness and enhanced migration of cancer cells [44].

Interestingly, dihydrotestosterone and testosterone treatment of vascular smooth muscle cells led to their secretion of biglycan and decorin modified with GAG chains of increased length [115]. Moreover, estrogens were shown to promote androgen-resistant PC cell growth. These cancer cells exhibit high expression of aromatase, leading to increased estrogen secretion. Estrogen, through ERα, enhances matrix metalloproteinase 12 (MMP12), ECM remodeling, and correlated PC invasion [116]. Therefore, enhanced secretion of endogenous estrogen, produced by increased aromatase levels, promoted the ERα-dependent expression of MMP12 expression, resulting in ECM remodeling in this PC model.

Another, SLRP, endocan, has also been implicated in PC. This PG is expressed by endothelial cells and has roles in angiogenesis. The expression of endocan is higher in metastatic PC-3 compared to non-tumorigenic PWR-1E PC cells. Downregulation of endocan in PC-3 cells decreased their migration and their production of the angiogenic CXCL3 chemokine [117].

### 4.3. PGs in the Process of Hormone-Dependent Cancer Metastasis

PGs have been implicated in the process of cancer metastasis [118]. Various cancer types, including prostate and breast, exhibit partiality for bone metastasis [119]. The bone microenvironment is unique, and upon colonization, becomes associated with progressive metastasis and high mortality. The bone, at first, presents a “hostile” niche for cancer cells, which is in continuoution reprogrammed by cancer cell-dependent factors into a “permissive” environment, e.g., reactive TME [120].

The expression of the HSPG perlecan is upregulated in prostate TME [121]. Furthermore, increased expression of MMPs results in the release of active perlecan fragments that can regulate essential PC cell functions such as adhesion and invasion [122]. Moreover, the release of growth factors bound to perlecan HS chains likewise contributes to various tumor-related processes, including angiogenesis [123]. Indeed, Cruz et al. suggest that modifications of perlecan-abundant TME, such as bone, “flip the molecular switch” and transform the “hostile” stroma into a permissive environment with the ability to facilitate cancer dissemination and metastasis [124].

Recent findings indicate that metastatic bone PC causes osteogenesis by orchestrating the recruitment and osteoblastic differentiation of mesenchymal stromal cells (MSCs) and is mediated by betaglycan, a cell surface PG, and a coreceptor for TGFβ [125].

Versican expression has been associated with adverse outcomes in prostate and breast cancer, including cancer relapse and poor patient prognosis [126]. Indeed, the versican G3 domain has been implicated in enhancing BC cell growth, migration, and bone metastasis through GFR/ERK or AKT signaling^119^. Part of the G3 effect was executed through downregulating pre-osteoblast cell growth and differentiation, which supports the generation of a permissive TME [127]. Recently, a novel Snail/PAPSS2/versican signaling axis has been identified in BC cell lung metastasis [128]. The transcriptional factor Snail is highly expressed in BC and promotes tumor relapse [129]. Snail enhances the expression of both the versican gene and the PAPSS2 gene; the latter encodes a sulfation pathway enzyme. A decreased ability of MCF7 and MDA-MB-231 BC cells to migrate was determined upon downregulation of PAPSS2 in these cells. Importantly, PPASS2-deficient MDA-MB-231-shPAPSS2 cells exhibited attenuated lung metastasis and decreased micrometastatic nodules in an animal model, whereas PAPSS2 overexpressing MDA-MB-231 cells exhibited increased lung metastasis. Indeed, the expression of Snail, PAPSS2, and versican is positively correlated in BC tissues [128]. Versican and tumor-associated macrophages (TAMs) have a synergistic effect on BC growth and metastasis. Thus, the deposition of versican to stroma is positively associated with the accumulation of TAMs in biopsies of advanced stage canine mammary carcinoma. Furthermore, TAM accumulation and versican expression in lung determined the number of metastatic pulmonary lesions [130].

Heparanase is an HS and heparin-degrading enzyme with multiple roles. By cleaving HS bound to the cell surface and ECM HSPGs, heparanase controls the localization and deposition of heparin-binding growth factors and cytokines, thus affecting various signaling pathways that regulate gene expression and cell functions [131]. Moreover, heparanase can activate nonenzymatic mechanisms that affect signaling at the surface of the cell, such as binding to TLR2 and -4, to regulate the macrophage inflammatory response [132]. A study utilizing the mouse mammary tumor virus (MMTV) to direct the expression of heparanase and the C-domain (8c) to the mammary gland epithelium of transgenic mice showed the participation of heparanase in BC metastasis. Indeed, these authors showed that the mammary gland branching morphogenesis is enhanced in MMTV-heparanase and MMTV-8c mice and was correlated with upregulated Akt, Stat5, and Src activation. Moreover, MMTV-heparanase mice are a permissive host for BC development as tumor growth and lung metastases increased. This study indicates that heparanase contributed by the TME has a crucial role in BC pathogenesis [133].

Several studies confirm the role of the HSPGs, syndecans, in the metastasis processes. For example, syndecan-2, a transmembrane PG, appears to act as a coreceptor for IGF-I. Suppression of its expression reduces IGF-I-dependent cell migration as this protein is essential for ERK1/2 phosphorylation, a well-known mediator of IGF-I signaling [35]. Furthermore, syndecan-2 is found to be highly colocalized with ezrin, a protein that connects membrane receptors to the cytoskeleton upon IGF-I treatment and thus facilitates the progression of IGF-I-dependent fibrosarcoma cell migration [35]. Importantly, the interplay of EGFR and IGFR is indicated as an essential factor in the tumorigenesis of BC [46,134,135]. These interactions are partly executed through PG action [134,135]. Indeed, a dichotomy of decorin effects on IGF-IR signaling was identified in normal as compared to cancer tissues [136].

Another study indicates that syndecan-1 silencing results in attenuated BC cell metastasis to the brain as migration across the blood–brain barrier and adhesion to the perivascular regions of the brain are reduced, while its overexpression has the opposite effect [137]. Of note, an inverse correlation was determined between syndecan-1 and ER expression in ER (α+) BC, whereas overexpression of syndecan-1 was identified in aggressive ERα-negative BC subtypes [138]. However, the effects of syndecan-1 seem to be hormone-dependent. Thus, epithelial syndecan-1 expression in BC is correlated with an ER-negative status, whereas the expression of syndecan-1 in stroma is associated with ER-positive subtypes [139]. Syndecan-1 downregulation increased fibronectin-dependent cell adhesion and migration of MDA-MB-231 ER-negative cells. This was correlated to the upregulated activation of β_1_-integrins. Furthermore, syndecan-1-deficient BC cells exhibited enhanced activation of focal adhesion kinase in a manner dependent on nuclear factor kappa-β (NF-κΒ) signaling and Il-6 expression [140]. This signaling axis was correlated to increased resistance of BC cells to irradiation [140]. Moreover, soluble and membrane-bound subtypes of syndecan-1 were shown to exert discrete effects at different phases of BC pathogenesis. Shedding of syndecan-1 due to enzymatic cleavage initiates a switch from a proliferative to an invasive phenotype in an MCF-7 BC cell model [141]. These results suggest that syndecan-1 could be a therapeutic target for BC [138] or utilized as a predictive marker of response to neoadjuvant chemotherapy [142].

Interestingly, it has been suggested that syndecan-1 could contribute to androgen-dependent to -independent conversion of PC. Thus, syndecan-1 expression is upregulated in androgen-independent PC cell lines DU145 and PC3 in comparison to androgen-dependent LNCaP PC cells. Moreover, when LNCaP cells are cultured under conditions of androgen deprivation, the expression of syndecan-1 increases. Downregulation of syndecan-1 expression in P3 xenografts resulted in decreased tumor size and tumor angiogenesis [143]. The above data demonstrate an intriguing connection between hormone action and effects exerted by syndecan-1 in BC and PC pathogenesis and could present a therapeutical target based on hormone response status.

**Figure 2:**
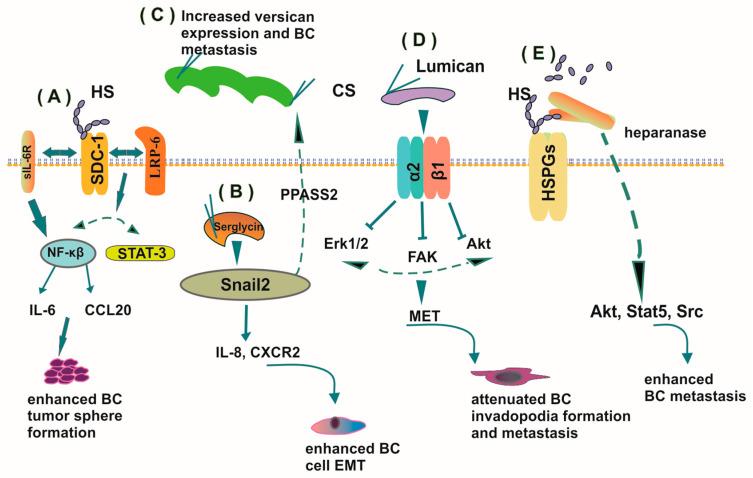
Roles of PGs in hormone-dependent cancer metastasis. (**A**) Syndecan-1, partly through interactions with sIL-6R and LRP-6, induces NF-κΒ and STAT3 signaling to affect Il-6 expression and CCL20 expression, resulting in BC cell adhesion, tumor sphere formation, and metastasis. (**B**) Serglycin, through autocrine activation of the IL-8/CXCR2 signaling axis, causes increased expression of mesenchymal markers vimentin, fibronectin, and epithelial-to-mesenchymal transition (EMT)-related transcription factor Snail2, which results in enhanced EMT of BC cells. (**C**) Snail enhances the expressions of both the versican gene and the PAPSS2 gene; the latter encodes a sulfation pathway enzyme to promote breast cancer (BC) metastasis and tumor relapse. (**D**) Lumican enhances the expression of α2 and β1 integrin subunits in aggressive BC cells that attenuate downstream signaling pathways, including FAK, ERK1/2, MAPK 42/44, and Akt, resulting in increased MET and downregulation of metastasis. (**E**) Heparanase, a heparan sulfate degrading enzyme, enhances Akt, Stat5, and Src signaling to upregulate BC metastasis.

A study in a murine mammary carcinoma model demonstrated that serglycin is essential for metastasis in lung, as serglycin-deficient cells present higher E-cadherin expression which is correlated to epithelial phenotype and cell–cell adhesion that attenuates cell migration [144]. These results are in agreement with the previous study in which Korpetinou et al. (2013) showed that BC cell lines highly express serglycin, which promotes migration and invasion, possibly via regulating actin cytoskeleton remodeling [145]. This hypothesis arises from its colocalization with actin in areas of cell–cell adhesion and filopodia-like structures in nonmigrating and migratory cells, respectively. The representative roles of PGs in hormone-dependent cancer metastasis are depicted in Figure 2.

### 4.4. The Role of PGs in Tumor-Dependent Angiogenesis

Cancer cells exhibit some unique capabilities that contribute to the malignant phenotype. These capabilities, including replicative immortality and resisting cell death, promote angiogenesis to provide the tumor tissues with the necessary nutrients [96]. Importantly, these properties can be affected by PG action. Normal vasculature is stimulated to develop tumor neovascularization through abnormal changes in the action of pro- or anti-angiogenic factors. GAG chains bound into PGs are capable of interaction with these factors or their receptors, enhancing intracellular signaling to induce vascular development [146]. Thus, domain I at the N-terminal of the angiogenesis-regulating PG, perlecan, is commonly substituted with three HS chains, whereas at the C-terminal, domain V can be decorated with HS and/or CS chains [147]. Indeed, the pro-angiogenic activity of perlecan is partly attributed to its interaction with the FGF receptors via the HS chains present at the N-terminal domain I [148]. Moreover, HS chains bound to perlecan domain V affect the VEGF–VEGFR2 axis to regulate developmental and tumor angiogenesis [149,150]. In continuation, however, perlecan was found to harbor anti-angiogenic properties as its cleaved C-terminus, endorepellin, inhibits angiogenesis [151]. Endorepellin is proteolytically cleaved from secreted perlecan through the action of cathepsin L [152] and exerts effects independent to intact perlecan [153]. Indeed, endorepellin bindsVEGFR2 and α2β1 to downregulate these receptors’ activities, resulting in angiostatic effects [154].

Syndecan-2, which is most frequently expressed in mesenchymal cells [35], seems to increase angiogenesis and control the death of subjected to chemotherapies PC cells [155]. High stromal syndecan-1 expression was found to enhance the proliferation of BC cells as well as to facilitate angiogenesis, being partly responsible for the higher mammographic density of tumor tissues [77]. Indeed, in MDA-MB-231 BC xenografts, the presence of fibroblasts enhanced tumor growth, which was further facilitated by the overexpression of syndecan-1. Moreover, Maeda et al. showed that the expression of stromal syndecan-1 in xenografts was positively correlated with increased microvessel density and vessel area. Importantly, the expression of syndecan-1 to stroma was correlated with both tumor growth and vessel density in BC patient biopsies [156]. Lumican was shown to inhibit angiogenesis in various tumors [7]; however, the utilization of the 4T1 BC model demonstrated lumican attenuated 4T1 tumor growth and lung metastasis, but not angiogenesis [157].

The type III TGFβ receptor (TGFβR3, betaglycan) has been attributed a role in tumor suppression in PC [158]. Indeed, the loss of betaglycan can also modulate the biological response of PC cells to testosterone and lead to the malfunction of growth factor pathways that regulate growth and angiogenesis [159]. Moreover, soluble betaglycan was shown to inhibit TGF-β-dependent angiogenesis in a PC model [160]. Recently, it was demonstrated that the seeding of tumor cells to the lungs facilitated the expression of SDC-4 by lung endothelial cells. These authors produced a CCL2 mutant protein fused to human serum albumin (dnCCL2–HSA chimera) which exhibits a high affinity to GAG chains. The dnCCL2–HSA chimera protein binds to lung endothelial cells due to its high affinity to HS, which strongly attenuated breast and prostate cancer cell transendothelial migration, among others [161].

The above evidence convincingly shows the involvement of PGs in processes related to the metastatic cascade of breast and prostate cancer. Moreover, a striking specificity of PG action between different subtypes of the tumors is indicated.

### 4.5. Xenobiotics and Hormone-Dependent Cancer-Implications of the ECM Niche

Xenobiotics are foreign chemical substances not produced in organisms or the environment and not expected to be present within the organism. They also cover substances that are naturally produced but are present in much higher concentrations than usual, or they are bio-transformed by enzymes in the organism [162]. Long-term exposure to either foreign, natural, or bio-transformed xenobiotics states may be the cause of cancer formation [163]. Cancer development through exogenous or endogenous agents involves DNA alterations that lead to mutations or genomic damage and modulation of cell function and signaling as well as changes in the host microenvironment that facilitate tumor progression and the acquisition of additional genetic events [164]. Xenobiotics and carcinogens, in general, influence cancer initiation, development, and maintenance, but most importantly, they can control therapeutic response and resistance.

BC and PC, the most common neoplasms, are estrogen-dependent tumors. Xenoestrogens (XEs), natural or synthetic, are endocrine-disrupting chemicals (EDCs) found in the environment, food, air, and cosmetics which interact with estrogen receptors and impact both metabolic pathways and the immune system [165,166]. Prolonged exposure to EDCs that interact with steroid receptors impacts the cellular mechanisms and, hence, can drive tumorigenesis. Hundreds of EDCs identified in the environment were correlated to BC incidence [167]. The ECM of each tissue can be modulated by the exogenous factors, resulting in a permissive microenvironment that leads to altered cell growth, adhesion, and migration. Other biochemical and metabolic consequences involve hypoxia and genetic instability [164,168,169,170]. Overall, there is a need for evaluation of the impact of xenobiotics on the ECM niche of TMEs [164]. Future studies should focus on determining changes in the deposition and composition of ECMs caused by xenobiotics, as well as in identifying signaling pathways that lead to carcinogenesis.

## 5. The Contribution of PGs to the Immunobiology of Hormone-Dependent Cancer

Cancer immunosurveillance is a complicated process influenced by the tumor’s cellular origin and anatomic location, type of transformation, inherent immunogenicity and stromal response, and cytokine production [171]. It is now apparent that tumor progression and its confrontation by the immune system are affected by the TME, which develops mechanisms to escape cancer immunosurveillance [172]. Indeed, the TME boasts different composition of cancerous, non-cancerous, stromal, and immune cells in each phase of cancer progression [1], producing different cytokines and chemokines and, finally, affecting cancer immunoediting [173]. Tumor cells can coexist with cells of both the innate and adaptive immune systems and produce some molecules necessary for macrophage recruitment and positioning in tumors. Analog alterations follow the changes in cellular effectors in ECM components [2,3]. The TME is thereby reminiscent of inflammatory conditions in healthy tissues [96]. The involution of the immune system in dealing with arising or already formed tumors consists of three different phases: elimination, connected to cancer immunosurveillance; an equilibrium that is the period of delay caused by the immune system after incomplete tumor destruction in the elimination phase; and escape, the final phase when a tumor has surpassed the immune system mechanisms of the elimination phase [2,171].

Questions regarding the importance of the endocrine and immune system interactions go as far back as 1995. At that time, it was established that growth hormone and prolactin are neuroendocrine hormones affecting immune system function and development [174], but what the mechanisms were, and how they can be therapeutically confronted required another decade of research to resolve [175,176].

Various environmental factors contribute to the disruption of these intricate mechanisms, among which the continuous exposure of humans to endocrine-disrupting chemicals (EDCs) can trigger estrogen-dependent tumors [166]. Moreover, due to the link between the endocrine and immune system, EDCs impact the potency of the immune elements leading to various dysfunctions [177]. In addition to the steroid-related system that can increase cancer risk, non-steroid substances (e.g., compounds associated with insulin resistance and inflammatory cytokines), can directly impact immune system functionality [178]. Hence, chronic inflammatory conditions [179] and the obesity phenotype [180] can be at the ground of carcinogenesis [181,182].

As stated, inflammation is frequently connected to tumor development and progression, as cells responsible for cancer-associated inflammation can induce malignant progression through the recruitment and activation of inflammatory cells, leading to immunosuppression [183]. ECM-mediated cues contribute to the inflammatory status of the TME [2,3]. The extracellular PG, biglycan, has a vital role in controlling inflammation [184] and is also reported to be a tumor promoter by regulating growth factor and cytokine signaling pathways [40,185]. In addition, biglycan, through interaction with TLR2/4 receptors on the surface of macrophages, promotes inflammation by triggering the synthesis of two cytokines important for cancer progression, TNFα and CCL2 [186,187], and thus enhances tumor growth in many cancer cells [68]. On the other hand, decorin acts as a pro-inflammatory molecule that interacts with TLRs and suppresses tumor growth [188].

Serglycin is expressed by all inflammatory cells and may regulate the biosynthesis, secretion, and targeted delivery of many inflammatory mediators as its role is to package numerous molecules and release them upon inflammation. Hence, it is evident that this intracellular PG enhances the inflammatory process and supports tumor growth and metastasis [26]. More specifically, in BC cells, serglycin promotes EMT and cell anchorage-independent growth, migration, and invasion [145]. Regarding hyalectans, some studies demonstrate versican participation in triggering inflammation. This molecule binds the TLR2 receptor on endothelial cells and fibroblasts, activates these cells and, finally, triggers the secretion of inflammatory cytokines [189].

The older and most validated cancer treatment is endocrine therapy. As some tumors are hormone-dependent and their actual growth relies on hormone stimulation, therapeutic interventions that aim to block hormone action have been successful [190]. The tumor growth is also dependent on growth factors, so depriving tumor cells of growth factors is an alternative option [135]. Nevertheless, clinical results have shown that hormone/growth factor deprivation is not adequate for successful therapy [190]. Hormones and growth factors regulate the immune system and jointly participate in homeostasis and pathological conditions [191].

It should be noted that the ECM acts as a reservoir for biologic mediators [2,43,192]. Thus, the different ECM proteins form and remodel complex nets, producing compartments which topically regulate the bioavailability of active mediators that affect cancer cell growth, survival, migration, and invasion. Indeed, PGs, with their versatile structure, contribute significantly to the generation of hormone/growth factor concentration gradients in the ECM and, by extension, in the TME [193,194].

On the other hand, hormones and growth factors regulate PG expression. Thus, estrogen regulates the expression of syndecan-1, which acts as an antagonist of the ER and is overexpressed in hormone receptor-negative BC subtypes [138]. Moreover, estrogen regulates the expression of heparanase in carotid walls, whose enzymatic action intrinsically modulates the activities of heparin-binding growth factors [164] and, by extension, angiogenesis [131].

The effects of steroids on immune cells have been studied in many diseases [195,196], and there are apparent differences between sexes. Thus, innate immune cells function through activation of pattern recognition receptors (PRRs), and their expression differs by the sex [197], e.g., induction of TLR7 signaling in women induces higher production of interferon-α compared to men [198].

### 5.1. Androgens and Estrogens Influencers of Immune Cells

The immune system has sex particularities, and though the reasons are still under study, it is most probably the case that sex hormones and sex hormone receptor-mediated events play an essential role in determining these differences [199].

Estrogens can influence T cell function in a concentration-dependent manner, favoring an anti-inflammatory response. The Th1 type immune response appears at low estrogen doses, while high doses induce a Th2 response. Estrogens reduce IL-6 secretion and STAT3 activity but upregulate IL-10, CTLA4, and PD-1 in regulatory T cells. In animal models of BC treated with ER antagonists, reduction of suppressive CD4+ CD25+ Treg activity was shown [200]. Estradiol levels within the PC tumor microenvironment can stimulate inflammation-related processes [201,202]. Estrogen induces interferon-gamma and nitric oxide production in lymphocytes [203]. On the other hand, altered levels of various inflammatory molecules can mediate aromatase expression and estrogen secretion and increase BC risk [204].

Androgens suppress the activity of immune cells by increasing synthesis of anti-inflammatory cytokine mediators, like IL-10, and repressing nuclear factor κB (NF-κB), cJun, and IRF1 or IL-12 secretion [205,206]. Furthermore, androgens reduce the phosphorylation of STAT4 and inhibit Th-1 lymphocyte polarization. Moreover, it was reported that Th2 lymphocytes could produce steroids, decreasing T-helper cell activation and inducing Treg activation [207,208]. The mechanisms of immune cell interaction with hormones may lead to new immunotherapies in hormone-dependent cancers.

**Figure 3:**
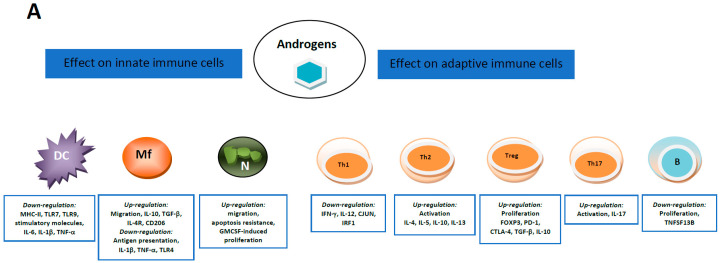

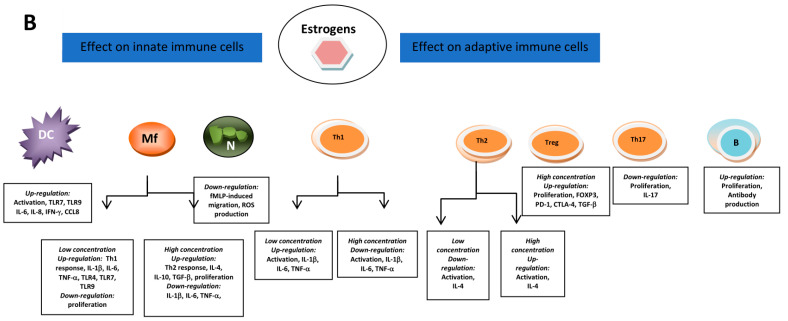
Effect of androgens and estrogens upon innate and adaptive immune cells. (**A**) Androgens mainly activate innate immune cell migration, apoptosis resistance, and inhibit antigen (Ag) presentation in correlation with downregulation of MHCII and costimulatory molecules. Androgens induce inhibition of Th1 and activation of Th2, Treg, and Th17 while downregulating B lymphocytes. (**B**) Estrogens induce various effects on innate immune cells depending on the estrogen dose; for macrophages (Mf) at low doses, Th1-type is induced, while at high concentrations, a Th2-type response is induced. Estrogens mainly activate dendritic cells (DC) while they exert inhibitory effects on neutrophils (N). Estrogens affect adaptive immune cells in a dose-dependent manner; at low concentrations, they activate Th1 and Th2, while inhibiting them at high concentrations. At high estrogen concentration, Treg are activated, Th17 inhibited, and B cells activated. THFS13B—*TNF superfamily member 13b*; IRF1—*interferon regulatory factor 1*.

Figure 3 summarizes the main effects induced by steroid hormones on innate and adaptive immune cells. The best-studied hormone-dependent cancers related to the immune system elements are breast and prostate cancer.

### 5.2. Breast Cancer—Immunological Perspective and Contribution of PGs

Mammary gland development and homeostasis are dependent upon the ovarian hormones progesterone and estrogen, and for pregnancy and lactation, upon prolactin. Importantly, HSPGs contribute significantly to the process of mammary gland branching morphogenesis but are also implicated in BC initiation [133]. Mammary development also has an immunological branch consisting of immune cells and cytokines that contribute to the mammary gland homeostasis and, upon deregulation, can lead to BC. Cytokine secretion and recruitment of various immune cells, like macrophages, eosinophils, mast cells, and lymphocytes, result in an inflammatory milieu, increasing the probability of BC initiation [209]. In this fine-tuned orchestra, where the immune system cues mingle with endocrine factors, RANKL and NF-κB are probably the main actors. Progestin and progesterone increase RANKL expression in the murine and human mammary gland, respectively [210,211]. These molecular machines, RANKL signaling, and NF-κB activation direct immune system elements toward carcinogenesis by undermining antitumor immunity and favoring chronic inflammation [212,213]. The Treg lymphocyte subpopulation expresses RANKL within BC tissue, and this overexpression is associated with pro-metastatic processes in Erbb2+ tumors [214]. Hence, there is an interplay between mammary epithelial cells and Treg cells that sustains cancer-associated inflammation.

Inflammatory breast cancer (IBC), the most aggressive form of breast cancer, represents approximately 2.5% of newly diagnosed breast cancers in the United States [215]. It has been shown that syndecan-1 is a modulator of the cancer stem cell (CSC) phenotype of triple-negative inflammatory breast cancer via the IL-6/STAT3, Notch, and EGFR signaling pathways [216]. Furthermore, the HSPG syndecan-1 was shown to exert an immunomodulatory role on the polarization of CD4+ T helper (Th) subsets isolated from the tumor tissues of patients with inflammatory BC and non-inflammatory BC [217]. These authors show that inflammatory BC exhibits a decreased basal frequency of Th1 and Th2 subsets compared to non-IBC. The downregulation of SDC1 increased the polarization of Th17 and Treg subsets of non-IBC and initiated the only Th1 subset polarization compared to control. Furthermore, it was suggested that tumor SDC-1 downregulation facilitates ex vivo polarization of CD4+ Th17 and Treg cells of non-inflammatory BC, possibly through increased expression of IL-23 and DLL4 [217].

Furthermore, heparan sulfate’s role in the regulation of infiltration of neutrophils and monocytes has been established in a dorsal air pouch inflammation model. Here, it was demonstrated that the degradation of HS chains of basement membrane PGs enhances the extravasation of inflammatory cells [218]. Another example of PG involvement in the formation of a “permissive” tumor microenvironment is the action of perlecan, whose inflammation-mediated modifications have the role of being a “switch” between tumor permissive and prohibiting states [124].

There are physiological fluctuations of estrogen and progesterone that can impact the local mammary gland immune system. During the luteal phase and pregnancy, the high concentrations of estrogen and progesterone impact the local immune system by recruiting immune cells via CSF1, TGFB, and IL10 [219], reducing the immune surveillance of the mammary epithelia. Progesterone reduces the phagocytosis of apoptotic cells, cells that release IL-4 and IL-10 immunomodulatory cytokines. Estrogen and progesterone direct macrophages into a tissue remodeling phenotype, downregulating MHC class II. In the mammary gland, there is a combined effect of estrogen and progesterone upon decreasing local immune surveillance, favoring immune tolerance and enhanced tumorigenesis [210]. These fluctuations can impact the expression of PGs like syndecan-1 which, in turn, can act as a coreceptor for growth factors and chemokines with the ability to modulate inflammatory processes [216].

The utilization of the nude experimental model of BC revealed further immune relations. Thus, Lindahl et al. have identified, using microdialysis, the cytokines that are released in the TME. Hence, animals treated with tamoxifen and fed with phytoestrogens like flaxseed (Flax) or mammalian lignan enterolactone (ENL) had decreased inflammatory IL-1β release and decreased microvessel density. Breast tumor stroma was found to be abundant in macrophages expressing the estrogen receptor. These authors conclude that in BC, macrophages and the immune proteins secreted by these cells have an essential role, and that these cells can be future targets for antiestrogen therapy [220].

Serglycin is highly expressed in the highly invasive, triple-negative MDA-MB-231 breast carcinoma cells [145]. This PG was shown to enhance interleukin-8 (IL-8) production in BC cells in a manner independent of their ERα status and facilitate their growth, invasion, and migration by initiating downstream IL-8/CXCR2 signaling consisting of PI3K, Src, and Rac activation. IL-8 is an inflammatory chemokine whose secretion is enhanced in various tumors, including BC [221]. IL-8 interacts with cell surface G protein-coupled receptors CXCR1 and CXCR2 [222] and, thus, triggers downstream pathways including for PI3K, MAPKs, and Rho-GTPases [223].

The immune system elements have lately become of great interest in the oncology domain. In various solid tumors, therapies that directly address immune mechanisms were approved and used [220]. The fact that the adaptive immune branch is involved in BC tumorigenesis has also triggered the quest for developing new immune therapies. Thus, cytotoxic T and T helper type 1 lymphocytes induce tumor eradication, while regulatory T (Treg) and T helper (Th) type 2 lymphocytes induce immune suppression that leads to pro-tumor processes. In this tumor milieu, hormone receptors and various molecules, including cytokines and growth factors, create a specific pattern that can be individualized among patients and can potentially be targeted by various immune therapies [222,224].

### 5.3. Prostate Cancer-Immunological Perspective

PC is highly dependent on androgens, and the biological effects of these hormones on PC initiation and progression are still subject to study to develop optimal treatment approaches [225].

In PC, as in the case of breast, cervical, endometrial cancers, the immune elements play an essential role. Immunity gains other patterns when the organism is aging, and this can explain the high incidence of PC in aged men. For example, antigen-presenting cells (APCs) that infiltrate the prostate tumor become tumor-associated macrophages in the prostate TME. This infiltration is linked with tumorigenesis and with patient clinical outcomes [226,227].

Androgens can favor an immunosuppressive state within the tumor, facilitating the immunologically ‘cold’ phenotype, while estrogens and progesterone effects are dose-dependent and can vary. Indeed, estrogens have been shown to induce the expression of HLA-DR and costimulating molecules in APCs [228]. In macrophages, on the other hand, low doses of estrogens upregulate the production of inflammatory cytokines (IL-1β, IL-6, and TNF-α), whereas at high concentrations, they reduce their secretion [229]. In the TME, androgens bind to their specific receptors expressed by various immune cells and induce distinct effects. Thus, testosterone reduces the inflammatory cytokine synthesis by macrophages [230], such as TNF-α, IL-1β, and IL-6, but increases CCL17 and CCL22 secretion [231]. Furthermore, testosterone reduces toll-like receptor 4 (TLR4) expression, attenuating the inflammatory phenotype of macrophages [232]. Androgens, likewise, decrease neutrophil functionality by reducing secretion of chemoattractant CXCL8 [233].

While immunotherapy has expanded in solid tumors, PC still relies on radiotherapy and androgen deprivation therapy (ADT). Recent studies have investigated the potency of NK cells to target specific PC cells. Thus, NK cells harvested from PC patients were primed with IL-2, in vitro, to kill PC3 (metastatic prostate cancer cell line) and K562. Around 50% of the patients developed NK cells that were active, in vitro, upon IL-2 stimulation, opening the possibility to use autologous NK cell-based immunotherapies in prostate cancer [234].

In animal models, a positive correlation was shown between the degree of immune suppression and the rate of tumor growth. In human tumor tissues, the presence of immune-suppressive elements, like Treg, can prognosticate the tumor’s aggressiveness. A better insight into the cellular effects of sex hormones within the TME can facilitate the development of new combination therapies. Importantly, PGs have been shown to contribute to the regulation of the above processes [2] and could present potential therapy targets/modulators. The combination of immune patterns of the tumor with the genetic particularities and sex steroid levels would probably create a biological framework for the integration of novel treatments.

## 6. Autophagy Affects Chemoresistance in Hormone-Dependent Cancers—Role of PGs

A common mechanism of resistance to cancer chemotherapy is autophagy, a catabolic process in which damaged organelles and protein aggregates are sequestered for degradation [235]. Furthermore, autophagy was shown to be required in the maintenance of cancer stem cells and strongly correlated to cancer therapy resistance [236]. Furthermore, stimulation of autophagy may suppress inflammatory responses that drive tumor growth and stop tumors escaping from the immune system [237].

However, even though numerous studies have focused on the role of autophagy in tumorigenesis, it is not well understood. It can be a pro-survival pathway used by tumor cells to increase their proliferation and development as well as to acquire resistance to cancer treatments [238]. On the other hand, autophagy can also be antitumoral and lead to cell death [239]. It seems that autophagy promotes regression in newly established tumors; however, it supports tumor progression in well-established tumors by maintaining cancer cell survival under stress conditions [239].

Several studies have demonstrated that chemotherapy-resistant BC cells exhibit increased autophagy [240,241]. The involvement of autophagy in BC treatment resistance is perpetrated through various mechanisms, including ROS reduction, apoptosis inhibition, or the maintenance of a dormancy-like protective state [242,243]. Thus, autophagy modulates the maintenance of B stem cells in autophagy-dependent BC cells through the regulation of IL6 secretion [244]. Likewise, the autophagy regulator ATG4A was shown to enhance the BC stem cell subset [245]. Moreover, it was shown that the resistance to lysosomotropic drugs for the treatment of retractive BC is correlated to autophagy with the involvement of CXCL5 [246]. These data have resulted in various clinical trials where autophagy mediators were therapeutic targets or biomarkers. Indeed, a different clinical response with discrete involvement of autophagy and apoptosis wad evident between chemoendocrine and endocrine therapy in BC [247]. Moreover, Ueno et al. showed that beclin 1 and LC3 autophagy marker expression was higher in BC cells upon exemestane treatment, whereas in pretreatment status, the expression of stromal beclin 1 was correlated with enhanced BC cell proliferation and inadequate clinical responses to neoadjuvant endocrine therapy [248].

Autophagy also increased chemoresistance in PC cells as AZD5363, an AKT inhibitor, in combination with chloroquine, a lysosomotropic autophagy inhibitor, induced apoptosis and postponed PC progression in animal models resistant to monotherapy [249]. Importantly, it was recently discussed that upon androgen deprivation in PC, several phenotypes of resistant PC cells are generated, correlated with autophagy [250].

PGs are implicated in the regulation of autophagy. Thus, decorin has recently been characterized as a “devouring proteoglycan” due to its autophagy facilitating properties [44]. Decorin, moreover, is well established to exert high-affinity binding interactions with receptor tyrosine kinases, facilitating receptor internalization/degradation and resulting in tumorigenic suppression [251]. Initially, decorin was found to induce, through VEGFR2 signaling, paternally expressed gene 3 (PEG3), previously characterized as a master regulator of macroautophagy in endothelial cells. Upon decorin stimulation, PEG3 relocalizes to BECN1- and microtubule-associated protein 1 light chain 3 alpha expression (LC3)-positive phagophores. Thus, decorin evokes a protracted autophagic program that is critically dependent on PEG3 expression [252]. Further, decorin inhibited anti-autophagic signaling via suppression of Akt/mTOR/p70S6K activity with the concurrent activation of pro-autophagic AMPK-mediated signaling cascades [253]. Of note, the VEGFR2/PEG3 pathway has, so far, been explicitly identified in endothelial cells. However, whether decorin can induce this pathway in cancer cells that express converging signaling machinery is a point that needs to be further investigated. This PG was found to be an autophagy-inducible protein in mouse cardiac tissue. Upon starvation, decorin was induced at the mRNA and protein level in vivo and in vitro, a process regulated at the transcriptional level by inhibiting the canonical mTOR pathway [254]. Importantly, DNC was shown to induce mitophagy in breast carcinoma cells via peroxisome proliferator-activated receptor γ coactivator-1α (PGC-1α) and mitostatin [255]. Systemic delivery of an oncolytic adenovirus expressing decorin induced, among other effects, mitochondrial autophagy in MDA-MB-231 breast cancer cells [256]. Decorin-mediated activation of autophagy seems to be connected with inhibited cell migration, as inhibition of autophagy and decorin knockdown reverses decorin anti-oncogenic action [257]. Versican G3 promoted cell apoptosis induced by C2-ceramide or docetaxel by enhancing the expression of pSAPK/JNK and decreasing the expression of GSK-3β (S9P). GSK-3β (S9P), correlated to autophagy, appears to function as a critical checkpoint in this balance of apoptosis and anti-apoptosis in BC [258].

Additionally, decorin participates in multiple signaling pathways known to regulate autophagy. Thus, epidermal growth factor receptor (EGFR)-mediated RAS/RAF/MEK/ERK signaling pathway plays a critical role in the induction of autophagy in various tumors [259]. Decorin is known to bind to EGFR and to activate the MAPK pathway, resulting in the induction of cyclin-dependent kinase inhibitor p21 and suppression of tumor growth [42]. Decorin-bound EGFR is internalized via caveolin-mediated pathways to reach lysosomes, where the receptor is degraded. Thus, local or systemic delivery of decorin can retard the growth of primary and metastatic carcinomas by reducing EGFR levels [260]. Moreover, p53, an essential regulator of autophagy [261], is a downstream mediator of decorin mediated cancer cell death [262]. The dissection of the role of decorin in autophagy could potentially be a crucial clue in efforts to exploit this pathway as a weapon for the chemoresistant BC.

It appears that biglycan steers signaling toward inflammation by interacting with CD14, whereas it can trigger autophagy by binding to CD44, both of which are TLR coreceptors [263]. Τherefore, biglycan has rightly been designated the role of a switch between inflammation and autophagy [44]. All these data confirm that PGs are essential molecules that participate in vital signaling pathways that regulate necessary cell behavior, and if they are dysregulated, they may exhibit special features that lead to malignancy.

## 7. Conclusions

The development of sensitive high-throughput technologies allowing the characterization of hundreds of matrix proteins has facilitated our understanding of the role of the TME and its ECM niche in homeostasis and disease [6,264]. It is now well established that mediators originating from the ECM have a crucial effect on all cellular functions implicated in cancer development and progression. Indeed, the ECM is the compartment of the TME, where the interactions among tumor and stroma cells are executed. In this review, we comprehensively discuss the roles of PGs, a major ECM component, in the complicated milieu of hormone-dependent cancer. As argued, PGs affect the bioavailability of active mediators, regulate the stiffness of the stroma immediately correlated to cancer cell invasion, and modulate the metastatic processes and angiogenesis. They can also act as regulators of the immune response and determine the response to therapy. All these functions are performed in a hormone-sensitive milieu where, importantly, both the expression and effects of PGs are discretely affected.

The complex interactions affected by the ECM need to be taken into account when designing efficient anticancer therapy. A remaining question is if and how can we interpose, therapeutically, to modify the ECM effects to facilitate the prognosis and survival of cancer patients. Significant advances that have been made in our understanding of the PG functions will soon allow the targeting of a specific protein and its downstream signaling pathways as a relevant pharmacological approach.

Some steps have been taken in this direction. CSPGs play a vital role in tumor growth, as negatively charged CS chains can interact with several different receptors and ligands, thus activating signaling pathways that stimulate tumor growth [265]. Modified CS, for example, and oversulfated CS chains have been established as potential anticancer agents [266]. Importantly, the coupling of liposomes by CS facilitated the uptake of the drug by MDA-MB-231 breast cancer cells, indicating that CS can be used as vectors for solid tumor targeting [267]. To obtain relevant data regarding the possibility of CS application as the basis for anticancer drug delivery systems, Park et al. chemically modified CS to prepare nanogels loaded with the anticancer drug doxorubicin [268]. The nanogels interacted with HeLa cells and were internalized together with the entrapped doxorubicin within the cytoplasm, probably via an endocytic mechanism exploited by sugar receptors.

Another example is the utilization of heparin, a sulfated polysaccharide, whose structure closely resembles the structure of HS chains bound into HSPGs [23]. It is widely used as a significant clinical anticoagulant due to its ability to bind with the serine–threonine protease antithrombin, causing the inhibitor to inactivate thrombin [269]. Sodium deoxycholate-conjugated heparin derivatives were used to prepare nanoparticles for in vivo tumor targeting and inhibition of angiogenesis based on chemical conjugation and the enhanced permeability and retention effect [270]. Obtained results confirmed that the conjugated heparin retained its ability to inhibit binding with the angiogenic factors, showing a significant decrease in endothelial tubular formation. Importantly, dendronized heparin–doxorubicin (heparin–DOX) conjugates showed potent antitumor activity, induced apoptosis, and had significant antiangiogenic effects in the 4T1 breast tumor model [271].

HSPGs and enzymes which regulate the modifications of HS chains are emerging as novel therapeutic targets in cancer [272]. Indeed, the enzyme heparanase has recently been characterized as an essential anticancer target [273]. It was shown that heparanase actively facilitates the metastasis of PC to the bone and other distant organs [274]. Moreover, the monoclonal antibody used for the treatment of BC, trastuzumab, increases fibronectin-dependent adhesion and attenuates invasion, growth, and angiogenic capacity of resistant to anoikis endothelial cells. This was correlated to changes in the expression of HS and HSPGs, including syndecan-4 and perlecan [275]. Another example of targeting PGs is the utilization of cationic amphipathic peptides with high affinity for HS bound into PGs at the cell membrane which displayed specific anticancer activity against PS in vivo [276]. Focusing on the specific properties of PGs and their activities at the crossroads among endocrine action, immune response, and the metastatic cascade introduces new directions in the ongoing battle against cancer.

Comprehensive studies in this field are resulting the better characterization of known as well as the discovery of new roles for PGs in cancerogenesis, which may lead to novel options for cancer diagnosis and treatment.
